# High-density single nucleotide polymorphism chip-based conservation genetic analysis of indigenous pig breeds from Shandong Province, China

**DOI:** 10.5713/ajas.20.0339

**Published:** 2020-10-19

**Authors:** Yanping Wang, Xueyan Zhao, Cheng Wang, Wenwen Wang, Qin Zhang, Ying Wu, Jiying Wang

**Affiliations:** 1 Shandong Provincial Key Laboratory of Animal Disease Control and Breeding, Institute of Animal Science and Veterinary Medicine, Shandong Academy of Agricultural Sciences, Jinan 250100, China; 2 Shandong Provincial Key Laboratory of Animal Biotechnology and Disease Control and Prevention, College of Animal Science and Technology, Shandong Agricultural University, Tai’ an 271018, China; 3 Shandong Xinji Animal Husbandry Co., Ltd, Tai’ an 271018, China

**Keywords:** Pig, Indigenous Breeds, Genetic Diversity, Population Structure, Conservation

## Abstract

**Objective:**

Shandong indigenous pig breeds are important Chinese pig resources. Their progressive population decline in recent decades has attracted attention towards their conservation. Conservation genetics of these indigenous breeds are essential for developing a conservation and utilization scheme.

**Methods:**

A high-density single nucleotide polymorphism (HD-SNP) chip-based comparative analysis of genetic characteristics was performed for seven Shandong indigenous pig breeds in the context of five Western commercial breeds.

**Results:**

The results showed that Shandong indigenous pig breeds varied greatly in genetic diversity, effective population size, inbreeding level, and genetic distance with the Western commercial breeds. Specifically, Laiwu and Dapulian displayed low genetic diversity, and had a genetically distant relationship with the Western commercial breeds (average F statistics [*F*_ST_] value of 0.3226 and 0.2666, respectively). Contrastingly, the other five breeds (Yantai, Licha, Yimeng, Wulain, and Heigai) displayed high genetic diversity within breed and had some extent of mixture pattern with the Western commercial breeds, especially Duroc and Landrace (*F*_ST_ values from 0.1043 to 0.2536). Furthermore, intensive gene flow was discovered among the seven Shandong indigenous breeds, particularly Wulian, Licha, and Heigai, as indicated by the large cluster formed in the principal component analysis scatterplot and small population differentiation (average of 0.1253) among them.

**Conclusion:**

Our study advances the understanding of genetic characteristics of Shandong indigenous breeds and provides essential information for developing an appropriate conservation and utilization scheme for these breeds.

## INTRODUCTION

Globally, China is the largest pork producing and consuming nation [1], with Shandong province, located in eastern China, being the fourth largest pig-producing region. Historically, several indigenous pig breeds have been developed by locals to meet their pork demands. In total, Shandong has seven officially authorized indigenous pig breeds (Laiwu, Dapulian, Licha Black, Yantai Black, Yimeng Black, Wulian Black, and Zaozhuang Heigai), six of which are recorded in national or provincial animal genetic resources [2,3] with the seventh (Zaozhuang Heigai) being newly approved (Announcement No. 168 of the Ministry of Agriculture and Rural Affairs of the People’s Republic of China). These indigenous pigs are well adapted to local environments, and some of them display prominent characteristics. For example, Laiwu pigs have excessively high level of intramuscular fat content (~10% in Laiwu vs ~1.5% in Yorkshire or Duroc×Landrace×Yorkshire) [4,5]; Dapulian pigs display higher resistance to porcine reproductive and respiratory syndrome than Duroc×Landrace×Yorkshire [6,7]; Licha Black is characterized by more vertebrae (1 to 2) compared with other Chinese native breeds [8].

Over the last few decades, more attention was paid to growth performance and lean meat content due to changes in consumer preference. Resultantly, Western commercial pig breeds (Duroc, Yorkshire, Landrace, Pietrain, and Berkshire) were introduced in China to increase productivity for these traits. This led to the progressive replacement and marginalization of Chinese indigenous pig breeds, some of which are currently close to extinction due to dwindling population numbers or hybridization with highly productive breeds [9]. Population decline of these indigenous breeds may lead to loss of allelic variation and reduced response to changing environments, especially to newly emerging pathogens [10, 11]. Thus, it may finally result in the loss of valuable future breeding resources. In addition, these traditional breeds are usually associated with local forms of pig husbandry forms, and their meat is used to produce high-quality products [5, 12]. Efficient conservation and utilization of local breeds are needed to support the development of sustainable pig industry. A comprehensive study of their genetic characteristics is a preliminary step for developing a conservation and utilization scheme. The genetic diversities of Shandong indigenous pig breeds have previously been evaluated using microsatellite markers [13] and mtDNA sequences [14]. Recent advances in high-throughput genotyping technologies, mainly high-density single nucleotide polymorphism (HD-SNP) chip and next-generation sequencing, have markedly facilitated studies on genetic characteristics at the genomic level, significantly extending our understanding of major questions in conservation genetics, including genetic distance, effective population size (*Ne*), and inbreeding level. For instance, the *Ne* and inbreeding coefficient are of major interest in conservation genetics, and common estimation methods based on pedigree information are sometimes not feasible due to the frequent unavailability of pedigree information in the indigenous breeds. High-throughput genotyping technologies allow the study of linkage disequilibrium (LD), and runs of homozygosity (ROH) [15–17] which enable us to estimate *Ne* and inbreeding coefficient. These technologies have been used to study other indigenous pigs [18–20], and studies have demonstrated their competency in assessing major questions in conservation genetics. Although high-throughput genotyping technologies provide a useful tool for genetic study, only one study has been recently conducted to investigate genetic diversity in Shandong indigenous breeds by using specific-locus amplified fragment sequencing (SLAF-seq) and a small sample size of seven to ten animals per breed [21]. Use of HD-SNP chip has the advantages of lower cost and higher efficiency in genotyping and statistics compared to high-throughput sequencing. Thus, more samples per breed can be genotyped to obtain a suitable representation of each breed using the SNP chip.

Here, HD-SNP BeadChips were employed to investigate the genetic characteristics of seven indigenous pig breeds from Shandong province, China, in the context of widely used Western commercial breeds. First, parameters for the genetic diversity and inbreeding within breeds were calculated. Second, the LD patterns and Ne were analyzed. Finally, individual clustering based on principal components (PCs) and historical admixture patterns were assessed to evaluate the structures among the analysed breeds, and genetic differentiation and phylogenetic trees were conducted to assess the relationship among them. All these joint analyses would provide valuable information for developing conservation and utilization strategies.

## MATERIALS AND METHODS

### Ethics statement

All animal procedures used were reviewed and approved by the Institutional Animal Care and Use Committee of Institute of Animal Science and Veterinary Medicine, Shandong Academy of Agricultural Sciences under permit number IACC20060101.

### Sample collection and SNP genotyping

A total of 397 pigs from seven indigenous breeds (Laiwu, Dapulian, Licha Black, Yantai Black, Yimeng Black, Wulian Black, and Zaozhuang Heigai) and five Western commercial breeds (Yorkshire, Landrace, Duroc, Berkshire, and Pietrain) were used in the study. The sample sizes of each analysed breed are presented in Table 1, and characteristics and pictures of the seven indigenous pig breeds are provided in the Supplementary File S1 of the supporting information. For each breed, all boars and unrelated sows (no common ancestry for three generations) in the conservation or breeding farms were sampled to obtain representative samples for each analysed breeds. The sample size per breed ranged from 26 for Heigai to 42 for Laiwu and Berkshire (Table 1). Genomic DNA was extracted from ear tissues using a routine phenol/chloroform method [22], and was diluted to a final concentration of 50 ng/mL.

All animals were genotyped using Porcine SNP55K Bead Chips according to the manufacturer’s protocol. The BeadChip was designed and promoted by Compass Biotechnology Corporation, Beijing, China (http://www.kangpusen.com/), and manufactured by Illumina (San Diego, CA, USA). Using PLINK v1.90 [23], SNPs were filtered with call rate ≥95% and minimum allele frequency (MAF) ≥0.05. The SNP positions within a chromosome were based on the current pig genome assembly (Sscrofa11.1). The Plink format genotype data have been deposited in Figshare (https://figshare.com/) with DOI:10.6084/m9.figshare.12765461 for ped file and 10.6084/m9.figshare.12765656 for map file.

### Genetic diversity

The following parameters of genetic diversity were calculated for each SNP and each breed using PLINK v1.90 [23]: MAF, observed heterozygosity (*H_O_*), expected heterozygosity (*H_E_*), IBS distance (*D*_ST_). Averages of MAF, *H_E_*, *H_O_*, and genetic distance (*D*, *D*=1–*D*_ST_) per breed were calculated.

### Runs of homozygosity

The ROHs were identified using autosomal SNPs passing the quality control and PLINK v1.09 [23]. We referred to the previous studies listed in the review [15] and the following criteria were employed in the analysis. The ROHs were defined by a minimum of 40 homozygous SNPs, length of 1,000 kb, minimum SNP density of one SNP/1,000 kb, and the largest possible gap between SNPs of 1 Mb. One heterozygous SNP and a maximum of five missing markers per ROH were permitted in the analysis. The number of ROH segments and the total length of ROHs in each individual were calculated and their mean estimated for each breed. Furthermore, the genomic inbreeding coefficient based on ROH (*F*_ROH_) was calculated for each individual as described by McQuillan et al [24], i.e. *F*_ROH_ = ∑L_ROH_/L_AUTO_, where ∑L_ROH_ refers to the total length of ROH and L_AUTO_ is the length of the autosomal genome covered by SNPs.

### Linkage disequilibrium analysis and effective population size

The LD was determined using the squared correlation coefficient (*r*^2^) between two SNPs for all marker pairs less than 5Mb for each breed independently using PLINK v1.09 [23]. To evaluate the LD pattern along chromosomes, the data were sorted into groups based on pair-wise marker distances, defined every 0.01 Mbp until 0.05 Mbp and every 0.05 Mbp until 1 Mbp, and *r*^2^ was then averaged for each group. Average *r*^2^ values for each breed were plotted against physical distance using the R program.

*Ne* was calculated for each breed using the following equation: *Ne* = (1/4c)×(1/*r*^2^–1) [25], where c is the genetic distance between two SNPs expressed in Morgans and *r*^2^ is the LD of different distances. The average high-density recombination rate reported by Tortereau et al [26] was considered to lead more correct estimates of *Ne*. The current *Ne* was calculated using the mean value of *r*^2^ for all 1 Mb bins across the entire genome as described by Herrero-Medrano [18], whereas the past *Ne* at generation T, where T = 1/2c, was estimated using the above equation [27].

### Population structure and relationship analyses

Principal component analysis (PCA) was performed using PLINK v1.09 [23], and the scatterplot of the first and second PCs was constructed to visualise clusters formed by individuals belonging to the same population. The program admixture version 1.3.0 [28] was used to examine historical admixture patterns of the analysed breeds. Cross-validation (CV) errors were calculated for each K value to identify the K value with the best predictive accuracy. F statistics (*F*_ST_) values were estimated using PLINK v1.90 [23] based on Weir and Cockerham’s formula [29]. Additionally, pair-wise evolutionary distances among the breeds were calculated using the following options, variance estimation method: none, substitution model: Tajima-Nei model [30], rates among sites: uniform rates, pattern among lineages: homogeneous, gaps/missing data treatment: pairwise deletion. Evolutionary distances were visualised by constructing the neighbour-joining (NJ) phylogenetic tree [31] using MEGA v7.0 [32].

## RESULTS

### Genetic diversity and inbreeding

After quality control, 39,300 were remained out of the 43,832 SNPs contained in the BeadChip. A summary of the SNP distribution and average distance of the adjacent SNPs on every chromosome are shown in Supplementary Table S1 of the supporting information. The physical distance between SNPs per chromosome was an average of 65.99 kb, ranging from 61.28 kb (Chr.18) to 78.86 kb (Chr.X), demonstrating that consistent SNP distance exists on the BeadChip. Additionally, the number of SNPs in different MAF ranges was also counted for each breed (Supplementary Table S2). The informative SNP (SNP with MAF >0.05) rates averaged 0.88 and 0.82 for Shandong indigenous breeds and Western commercial breeds, respectively.

The genetic diversity parameters estimated for each breed are presented in Table 1. The genetic diversity parameters varied considerably among the 12 analysed breeds. Laiwu displayed the lowest genetic diversity, with MAF, *H_O_*, *H_E_*, and *D* values of 0.1621, 0.2375, 0.2252 and 0.1855, respectively. In contrast, Yantai, Licha, and Wulian exhibited the highest genetic diversity, with Yantai displaying the highest MAF (0.2901) and *H_E_* (0.3750), while Licha and Wulian the highest *H_O_* (0.3837) and *D* (0.2981), respectively. Comparatively, all Shandong indigenous breeds, except Laiwu and Dapulian, displayed higher genetic diversity than the five Western commercial breeds.

ROHs for the analysed populations were estimated to de termine the inbreeding levels. As shown in Table 2, Wulian displayed the lowest number of ROH (16.17), the shortest length of ROH (129,121.27), and the lowest *F*_ROH_ (0.0527). Contrastingly, Duroc had the highest number of ROH (74.13), longest total length of ROH (681,349.23), and highest *F*_ROH_ (0.2783). Western commercial breeds had higher *F*_ROH_ than Shandong indigenous breeds (average 0.2134 vs 0.1312), while Shandong indigenous breeds had larger standard deviation than Western breeds (average 0.0732 vs 0.0441). Thus, our results show that Shandong indigenous breeds have lower averaged breeding levels. However, Shandong indigenous breeds have higher inbreeding variation within breeds compared to Western breeds.

### Linkage disequilibrium pattern and effective population size

To assess LD patterns, *r*^2^ was estimated for all SNP pairs in a distance up to 5 Mb apart across the genome, and the levels of LD at different distances for each breed are provided in Supplementary Table S3. Overall, Shandong indigenous breeds had lower *r*^2^ than Western commercial breeds. The averaged *r*^2^ values were 0.32, 0.23, and 0.17 for adjacent SNPs, SNPs 1 Mb apart, SNPs 5 Mb apart for Shandong indigenous breeds, and 0.44, 0.30, and 0.19 for Western commercial breeds, respectively.

**Figure 1:**
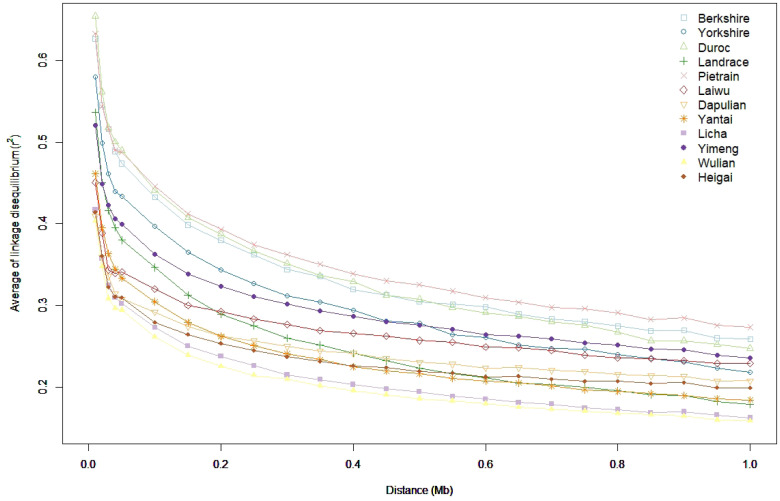
Linkage disequilibrium plotted against distance between single nucleotide polymorphisms across the 18 autosomes for the analysed pig breeds. BK, Berkshire; D, Duroc; L, Landrace; Y, Yorkshire; PT, Pietrain; LW, Laiwu; DP, Dapulian; YT, Yantai; LC, Licha; YM, Yimeng; WL, Wulian; HG, Heigai.

The LD along physical distance between markers was also plotted to visualise the LD along chromosomes, and the plot is presented in Figure 1. All breeds showed the similar trend of the average *r*^2^ with distance, *i.e.*, it decreased rapidly over the first 0.2 Mb distance, and then decreased gradually over the remaining 0.8 Mb distance. The LD of Landrace decreased by the half at 0.25 Mb, showing the highest LD decay, while the LD of Laiwu and Dapulian decreased by the half at 0.95 Mb, showing the highest LD persistence. Similar *r*^2^ values for all the distances were observed for Licha and Wulian, indicating the genetic closeness of the two breeds.

The present *Ne* was estimated for each breed based on the estimated *r*^2^ (Table 1). The present *Ne* of the 12 breeds analysed ranged from 69 to 129, with an average of 96.59. Generally, Shandong indigenous breeds had larger *Ne* than Western commercial breeds, with an average of 109.9 compared to 77.95. Among Shandong indigenous breeds, Wulian had the largest *Ne* (136), followed by Licha (130), while Yimeng had the smallest *Ne* (83), followed by Laiwu (92). The past *Ne* was also estimated. As shown in Supplementary Table S4, the past *Ne* of Shandong indigenous breeds was larger than those of Western commercial breeds, too.

### Population structure analysis

**Figure 2:**
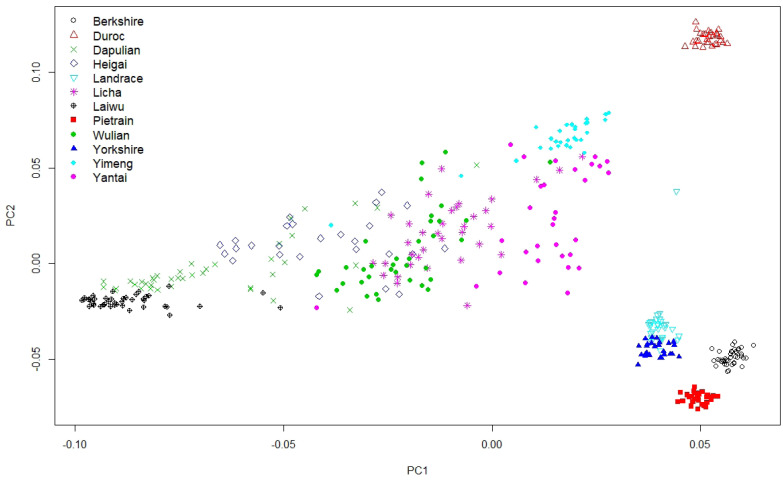
Population structure of the analysed pig breeds revealed by principal component analysis. BK, Berkshire; D, Duroc; L, Landrace; Y, Yorkshire; PT, Pietrain; LW, Laiwu; DP, Dapulian; YT, Yantai; LC, Licha; YM, Yimeng; WL, Wulian; HG, Heigai, PC1, the first principal component; PC2, the second principal component.

Firstly, PCA was performed to explore the clustering of individuals of different breeds (Figure 2). The results indicated that 89.59% of the total variance was explained by the first three PCs (53.06% by PC1, 20.30% by PC2, and 16.23% by PC3). As shown in Figure 2, visibly separated clusters were observed for the commercial breeds, Berkshire, Duroc, and Pietrain, whereas separated but slightly overlapped clusters were observed for Landrace and Yorkshire. This suggested that Western commercial breeds had distinct population structures. In contrast, a single large cluster was formed by the seven Shandong indigenous breeds. Laiwu and Dapulian slightly overlapped at the left end of the large cluster, and Yimeng and Yantai were located at the right end, close to the clusters of Western commercial breeds, particularly Duroc and Landrace. Licha, Heigai, and Wulian completely overlapped in the middle, indicating the close relationship among them. The areas of the seven pig breeds distributed are not far away and have had frequent economic and social exchanges over the history. Thus, intensive gene flow may have occurred among them.

**Figure 3:**
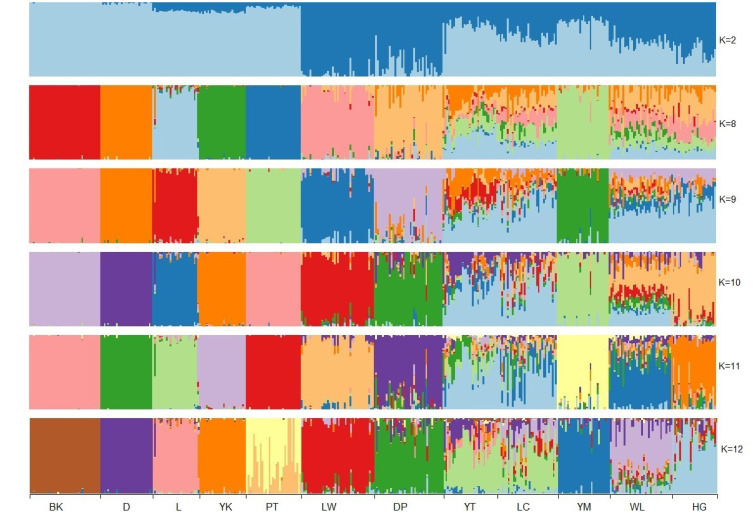
Historical admixture patterns of the analysed pig breeds. BK, Berkshire; D, Duroc; L, Landrace; Y, Yorkshire; PT, Pietrain; LW, Laiwu; DP, Dapulian; YT, Yantai; LC, Licha; YM, Yimeng; WL, Wulian; HG, Heigai. Each colour represents the proportion of the genome assigned to each assumed cluster.

Then, historical admixture patterns of the analysed breeds were assessed using K values from 2 up to 12 with Admixture. The CV errors (Supplementary Table S5) decreased as the K value increased, with a rapid decrease when K ranged from 2 to 8. The population admixture patterns with K = 2 and K ranging from 8 to 12 are shown in Figure 3. The Western commercial breeds formed one large cluster (marked in light blue) when K = 2, while Laiwu and Dapulian formed another one (indicated in dark blue). Laiwu, Dapulian, and five Western breeds appeared as differentiated clusters when K = 8, while the other five Shandong breeds shared the same cluster. Yimeng, Heigai, and Wulian were separated as distinct clusters at K = 9, 10, and 11, respectively, with an increase in K value. However, no noticeable differentiation between Yantai and Licha appeared with K values up to 12, suggesting that considerable admixture existed between them.

### Population relationship analysis

Pairwise *F*_ST_ values were further estimated to determine the extent of population genetic differentiation, and the results are shown in the lower diagonal of Table 3. High *F*_ST_ values were observed between Shandong indigenous breeds and Western commercial breeds, with an average of 0.21, indicating considerable genetic differentiation between them. Laiwu had the largest genetic differentiation with all the Western commercial breeds (0.2988 to 0.3489), followed by Dapulian (0.2411 to 0.2875). In contrast, *F*_ST_ values among Shandong indigenous pig breeds changed markedly from 0.0441 (Wulian and Licha) to 0.2554 (Laiwu and Yimeng), and most of their values were low with an average of 0.1253. Notably, Laiwu also displayed the highest genetic differentiation with the other Shandong indigenous breeds, especially with Yimeng (0.2554) and Yantai (0.2265).

**Figure 4:**
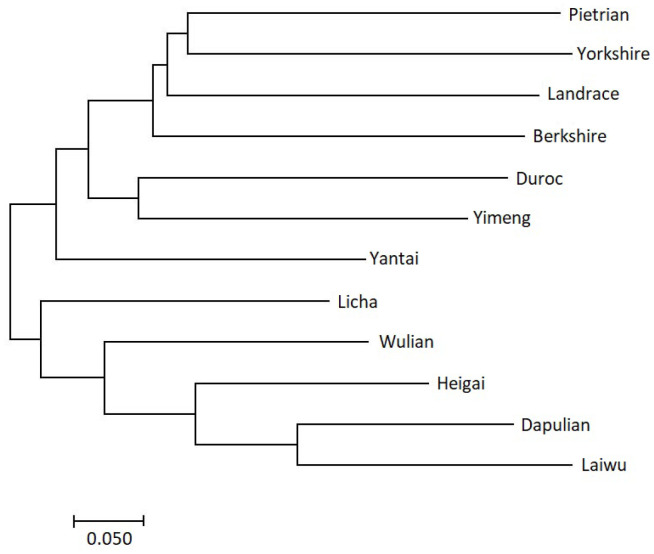
Neighbour-joining phylogenetic tree of the analysed pig breeds based on the evolutionary distance.

Finally, pair-wise evolutionary distances among the analysed breeds were calculated (the upper diagonal of Table 3) and visualised using an NJ phylogenetic tree (Figure 4). Generally, the evolutionary distances corresponded with the *F*_ST_ values. The Western commercial breeds clustered together at the top of the tree, while the seven Shandong breeds clustered together at its bottom. However, the clades formed by Shandong indigenous and Western commercial breeds were not completely separated. Yimeng and Yantai clustered first with Duroc before clustering with the other Shandong indigenous breeds, indicating the close relationship between the two breeds with Western commercial breeds, especially with Duroc.

## DISCUSSION

The progressive population decline of Shandong indigenous pig breeds has called for attention towards their conservation. Here, we conducted an analysis of the conservation genetics for the Shandong indigenous pig breeds based on HD-SNP BeadChip, Porcine SNP55K, to provide essential information for the sustainable protection and use of these genetic resources.

Porcine SNP55K BeadChip used in the present study was designed for genetic research of Chinese local breeds, and SNPs found in the Chinese local breeds were considered in it to improve the coverage of chromosomes and MAF of Chinese local breeds. The total SNP of the chip was lower compared to widely used Illumina 60K BeadChips [33] and its improved versions. However, all the SNPs could be unambiguously mapped to the current pig genome (Sscrofa11.1) with an even distribution. The results of our study demonstrated that the informative SNP rates with MAF >0.05 were more consistent among breeds, with an average of 0.87 and 0.80 for Shandong and Western breeds, respectively. In a previous study conducted in 304 Chinese and Western pigs using Illumina Porcine SNP60 BeadChip, severe SNP bias was observed between these pigs for a common subset of 15,911 SNPs with MAF >0.2, with 0.87 to 0.95 and 0.67 to 0.98 polymorphic SNPs for Western and Chinese breeds, respectively. However, no informative SNP rates with MAF >0.05 were displayed [19]. Thus, the Porcine SNP55K BeadChip SNPs are more suitable for genetic studies of Chinese indigenous breeds than those of Illumina Porcine SNP60 BeadChip.

Our results indicated genetic diversity varied largely among Shandong indigenous breeds. Laiwu and Dapulian were smaller than the Western commercial breeds, while the other five breeds were larger than the Western commercial breeds. Our results are consistent wholly or partly with the previous studies based on microsatellite [13], and SLAF-seq [21], but contrary to those based on mtDNA sequence [14, 34]. Wang et al [14] sequenced a control region sequence and observed that genetic diversity of Laiwu and Dapulian were comparable with that of Western commercial breeds. Quan et al [34] analysed the mtDNA hypervariable regions of 70 Chinese native pig breeds and showed that most native pig breeds have low genetic diversity, especially for those distributed in the Sichuan and Shandong Provinces. The inconsistencies between the genome sequence and mtDNA sequence results could be attributed to mtDNA being maternally inherited in sexually reproducing organisms, including pigs [35,36]. Also, a small segment sequence was analysed in the mtDNA based studies. These results were indicative of the influence sample size and representation has on research results.

Western commercial pigs have undergone intensive selec tion over decades, which has resulted in their reduced genetic diversity, high LD, and inbreeding [37,38]. Unlike Western commercial pig breeds, Chinese indigenous pigs have not undergone the intensive selection, and they are assumed to sustain higher levels of genetic diversity. The inconsistencies in our study could mainly be attributed to the small population size of the two breeds; only a few founders were left at the beginning of the preservation programs. Currently, several Western commercial breeds have been dominantly used to produce pork in the Chinese pig industry, and only a limited number of pigs from each breed are raised for conservation or high-quality product production. Small population sizes have probably led to some genetic variability loss.

Inbreeding levels and Ne are two essential factors in pop ulation protection. Traditionally, they have been evaluated based on pedigree information, which is however incomplete or completely unavailable for most indigenous breeds. Several methods, independent of the pedigree, have been developed to estimate inbreeding and *Ne* based on genomic data. *F*_ROH_, defined as the percentage of the genome covered by ROH, is regarded as an indicator reflecting the recent inbreeding history of a population and has also shown a good correlation with pedigree inbreeding coefficients [15,39]. *Ne* is estimated based on LD patterns and has been proven to be similar to that estimated based on the pedigree data [27,38]. Our results showed that breeds with high genetic diversity displayed low *F*_ROH_ and high *Ne* values. These congruous results demonstrated that the reliability in using *F*_ROH_ to indicate inbreeding and LD to estimate *Ne*.

Of the Shandong indigenous breeds, Laiwu presented the highest *F*_ROH_ (0.2263) value, even higher than most Western commercial breeds. The *F*_ROH_ values of the other Shandong breeds were low to moderate, with *Ne* above 100 except Yimeng (*Ne* = 83). Nevertheless, there was significantly large within-breed variation in *F*_ROH_ values for these breeds, indicating the presence of individuals with high inbreeding levels. Such individuals should be specifically considered when planning matings. Furthermore, an effective population size of 100 should be considered to maintain a population’s genetic diversity [40]. Some of Shandong indigenous breeds have *Ne* values slightly higher than the recommended number, while some are close to the recommended number. Thus, appropriate methods should be applied to control the inbreeding levels and improve *Ne* to protect these genetic resources.

All population structure and relationship analyses per formed in the present study demonstrated that Shandong indigenous pig breeds were genetically distant from the Western ones. Particularly, *F*_ST_ values, which are based on allele frequency differences among populations [41], quantitatively indicated the large genetic differentiation among these breeds. Shandong indigenous and Western commercial breeds had an average *F*_ST_ value of 0.21, ranging from 0.1043 to 0.3479. Based on the degree of genetic differentiation using *F*_ST_ thresholds (0.05, 0.15 and 0.25) specified by Hartl and Clark [42], moderate to high degrees of differentiation existed between them. These results are consistent with previous studies based on microsatellite and SLAF-seq [13,21].

Of the seven Shandong indigenous breeds, Laiwu and Dapulian had markedly high *F*_ST_ values with the Western commercial breeds, with averages of 0.3226 and 0.2666, respectively. Furthermore, Laiwu and Dapulian located at the distal end of the large cluster with slight overlap with other indigenous pigs (Figure 2), differentiated from Western commercial breeds when K = 2, and were firstly grouped together in the NJ trees (Figure 4). Collectively, these results indicated that Laiwu and Dapulian were less influenced by Western commercial breeds, being two indigenous pig breeds with unique genetic characteristics. Laiwu and Dapulian are distributed mainly in the middle of Shandong province, where traffic and economic development are relatively underdeveloped. In addition, their conservation farms were constructed earlier and in a better management compared to other Shandong indigenous breeds. Thus, they are better protected and less influenced by Western commercial breeds. These results are consistent with the results of previous studies based on mtDNA sequences [14], microsatellite markers [13] and SLAF-seq [21].

In contrast, the other five Shandong indigenous pig breeds had relatively small genetic differentiation with the Western breeds (*F*_ST_ values from 0.1043 to 0.2536). They were located close to Western breeds clusters (Figure 2) and had some extent of mixture pattern with the Western commercial breeds (Figure 3). All these results support that they have been influenced to some extent by the Western commercial breeds, especially Yimeng and Yantai. The five indigenous breeds are in the northeastern Shandong peninsula, where the traffic and economic development are relatively developed. Historically, western pig breeds were introduced to these districts in the early of 20th century and were used to improve the growth performance of the local breeds [3,8]. Additionally, there are relatively low pairwise *F*_ST_ values (0.0441 to 0.1489), overlapped in the cluster of PCA (Figure 2), and separated only when the K value was high (Figure 3). This suggested that extensive gene flow ever progressed among these breeds. Notably, the five breeds presented high genetic diversity, which may be attributed to gene flow among them as well as hybridization with the Western commercial breeds.

## Supplementary Information



## Figures and Tables

**Table 1:** Sample size, single nucleotide polymorphism, genetic diversity, and effective population size (*Ne*) of the analysed pig breeds

Populations	Sample size	MAF	Informative SNPs	*H_O_*	*H_E_*	*D*	*Ne*
Yorkshire	27	0.2491	33,851	0.3322	0.3269	0.2637	82
Duroc	30	0.2076	29,037	0.2719	0.2776	0.2261	69
Landrace	27	0.2580	35,214	0.3410	0.3367	0.2711	105
Berkshire	41	0.2004	28,374	0.2859	0.2683	0.2105	69
Pietrain	32	0.2184	30,340	0.3159	0.2906	0.2252	65
Laiwu	42	0.1621	25,648	0.2375	0.2252	0.1855	92
Dapulian	40	0.2025	31,801	0.2842	0.2779	0.2306	105
Yantai	31	0.2901	36,997	0.3791	0.3750	0.2979	112
Licha	35	0.2871	37,346	0.3837	0.3731	0.2973	130
Yimeng	30	0.2555	33,449	0.3497	0.3335	0.2632	83
Wulian	36	0.2856	37,709	0.3803	0.3732	0.2981	136
Heigai	26	0.2446	35,277	0.3553	0.3269	0.2611	112

MAF, minimum allele frequency; SNP, single nucleotide polymorphism; *H_O_*, observed heterozygosity; *H_E_*, expected heterozygosity; *D*, genetic distance within breed; *Ne*, effective population size.

**Table 2:** Characterization of runs of homozygosity of the analysed pig breeds

Breeds	Number of homozygous segments			Total length of homozygous segments			F_ROH_
		
	Mean±SD	Min	Max	Mean±SD	Min	Max	Mean±SD
Yorkshire	53.11±5.02	46	65	447,240.07±67,166.5	348,948	578,627	0.1827±0.0274
Duroc	74.13±6.75	55	87	681,349.23±85,465.98	540,502	957,542	0.2783±0.0349
Landrace	46.44±11.96	11	73	386,350.33±144,412.43	65,905.9	705,175	0.1578±0.0590
Berkshire	60.88±7.47	46	72	556,928.37±113,730.15	320,373	784,093	0.2275±0.0465
Pietrain	52.53±6.73	39	65	540,764.5± 129,453.81	379,068	905,718	0.2209±0.0529
Laiwu	54.55±19.22	12	81	554,027.29±240,477.84	69,173	1,111,670	0.2263±0.0982
Dapulian	34.03±11.64	7	54	439,235.03±230,602.04	38,055.4	990,579	0.1794±0.0942
Yantai	24.16±9.22	11	40	243,536.44±171,731.17	51,968.5	660,857	0.0995±0.0701
Licha	22.06±12.44	4	50	215,821.93±190,903.73	14,607.3	706,320	0.0882±0.0780
Yimeng	41.9±8.76	24	64	460,989.73±143,331.45	159,807	814,836	0.1883±0.0585
Wulian	16.17±7.36	2	39	129,121.27±115,407.34	10,303.2	678,825	0.0527±0.0471
Heigai	20.5±11.09	6	37	205,596.32±162,512.66	25,322.3	639,861	0.0840±0.0664

*F*_ROH_, genomic inbreeding coefficient based on runs of homozygosity (ROH); SD, standard deviation; Min, minimum; Max, maximum.

**Table 3:** Pairwise F statistics (*F*_ST_) (lower diagonal) and evolutionary distance (upper diagonal) values among the analysed pig breeds

	Yorkshire	Duroc	Landrace	Berkshire	Pietrain	Laiwu	Dapulian	Yantai	Licha	Yimeng	Wulian	Heigai
Yorkshire	-	0.6452	0.5534	0.5754	0.5422	0.7979	0.7473	0.5853	0.6171	0.6279	0.6251	0.6892
Duroc	0.2194	-	0.6210	0.5793	0.6357	0.9315	0.8109	0.5472	0.6047	0.5002	0.651	0.6994
Landrace	0.1345	0.1996	-	0.5423	0.5511	0.807	0.7535	0.5561	0.6031	0.6172	0.6234	0.6706
Berkshire	0.2102	0.2367	0.1835	-	0.5525	0.8362	0.7823	0.5726	0.6112	0.5824	0.6374	0.7029
Pietrain	0.1737	0.2409	0.1655	0.2216	-	0.8272	0.7571	0.5852	0.6225	0.6214	0.6433	0.6935
Laiwu	0.3064	0.3479	0.2988	0.3337	0.3263	-	0.3517	0.6135	0.5066	0.6473	0.4687	0.4108
Dapulian	0.2486	0.2843	0.2411	0.2875	0.2715	0.1074	-	0.5806	0.4997	0.6065	0.4671	0.4217
Yantai	0.1344	0.1449	0.1043	0.1812	0.1664	0.2265	0.1672	-	0.4947	0.5188	0.5197	0.5369
Licha	0.1496	0.1699	0.1291	0.1955	0.1823	0.1722	0.1245	0.0555	-	0.5264	0.476	0.4781
Yimeng	0.1791	0.1461	0.1617	0.2074	0.2085	0.2554	0.1965	0.0982	0.1043	-	0.5415	0.5568
Wulian	0.1506	0.1879	0.1361	0.2047	0.2265	0.1496	0.1031	0.0710	0.0441	0.1126	-	0.4548
Heigai	0.2031	0.2431	0.1838	0.2536	0.2338	0.1401	0.0998	0.1122	0.0799	0.1489	0.0625	-
