# Supplementary material for: High-density single nucleotide polymorphism chip-based conservation genetic analysis of indigenous pig breeds from Shandong Province, China
Source: Anim Biosci. 2020 Oct 19;34(7):1123–33. doi: 10.5713/ajas.20.0339 (PMC8255875; doi:10.5713/ajas.20.0339)
Supplement: Supplementary file 1 [file ajas-20-0339-suppl.pdf]

File S1. Characteristics and pictures of Shandong indigenous pig breed.

Laiwu pigs

Laiwu pigs are mainly distributed in Laiwu and its surrounding areas. The representative photos for male and female Laiwu pigs are shown in Figure S1. They are of medium size, drooping ears, straight lips and wrinkled forehead. Their coat is black with dense hair and long mane. They have more than 7 pairs of effective nipples, up to 12 pairs. Their growth rate is rather slow, and lean meat rate is about 40%. Sows of this breed have high prolificacy with the average litter size of 13.72. The most prominent characteristics of Laiwu is that they have extremely high intramuscular fat content (up to 10%).

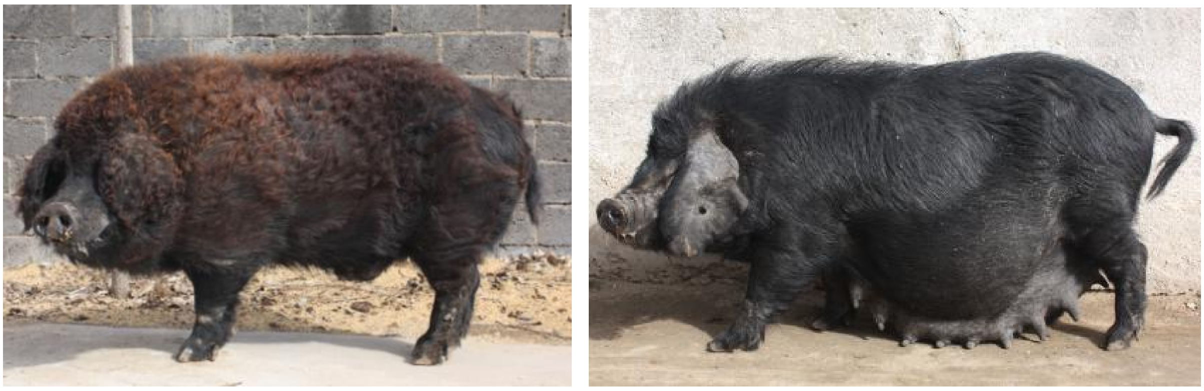

Figure S1. Pictures of Laiwu pigs (left:male; right:female) (The pictures were taken from reference 1 of the manuscript.)

Dapulian pigs

Dapulian pigs are mainly distributed in Jining and its surrounding areas. The representative photos for male and female Dapulian pigs are shown in Figure S2. They are of large size with drooping ears and lotus shaped forehead folds. Their coat is black and fluffy, and mane is thick and dense. They have more than 7 pairs of effective nipples. Their growth rate is slow, and lean meat rate is about 44%. Sows of this breed have high prolificacy with the average litter size 13.2. The most prominent characteristics of Dapulian is that they have strong disease resistance, especially to PRRV.

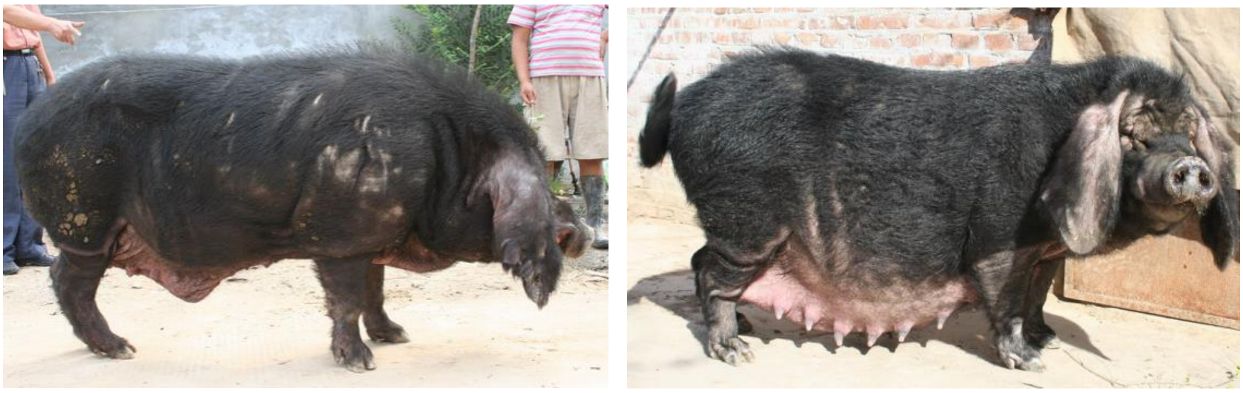

Figure S2. Pictures of Dapulian pigs (left:male; right:female) (The pictures were taken from reference 1 of the manuscript.)

Zaozhuang Heigai pigs

Zaozhuang Heigai pigs are mainly distributed in Zaozhuang and its surrounding areas. The representative photos for male and female Zaozhuang Heigai pigs are shown in Figure S3. They are of medium size, with plump hindquarters and large abdomen. The ears are of medium size, with half drooping. The mouth is wide, short and slightly cocked. There are irregular "八" shaped wrinkles on their forehead. They have more than 7 pairs of effective nipples, up to 10 pairs. The lean meat rate is about 46%. Sows of this breed have high prolificaty with the average litter size 12.85. Particularly, they have strong capability for digesting roughage.

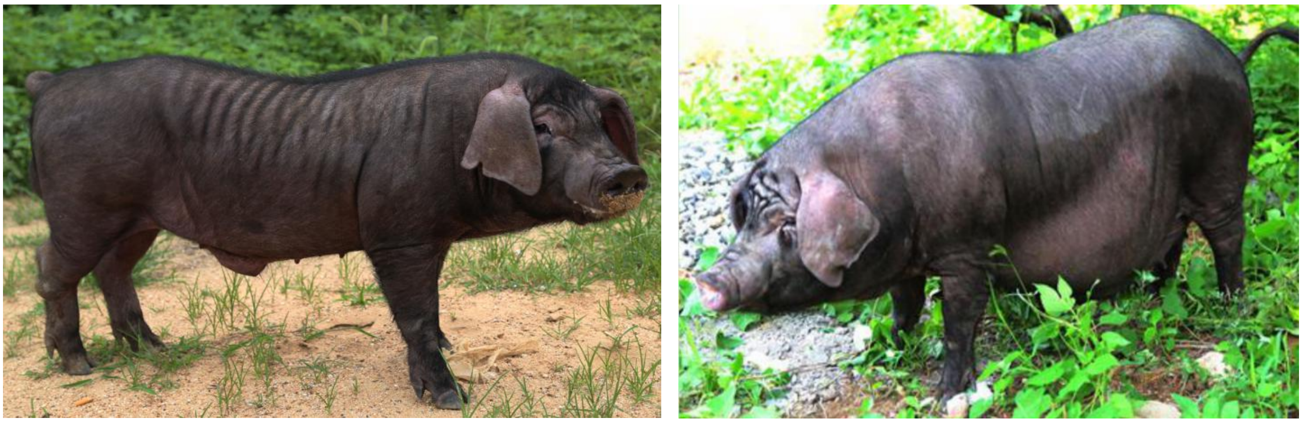

Figure S3. Pictures of Zaozhuang Heigai pigs (left:male; right:female) (The pictures were taken by us.)

Yimeng Black pigs

Yimeng black pigs are distributed mainly in Linyi and its surrounding areas. The representative photos for male and female Yimeng Black pigs are shown in Figure S4. They are of medium sized,

with broad and wrinkled forehead, medium-sized and forward leaning ears, and short and slightly pouted mouth. They have wide and deep chests, straight backs and waists and strong limbs. They have 7-9 pairs of effective nipples. Their growth rate is high compared with pigs of other Shandong indigenous breeds, and lean meat rate is about 50%. Sows of this breed have low prolificacy with the average litter size 10.

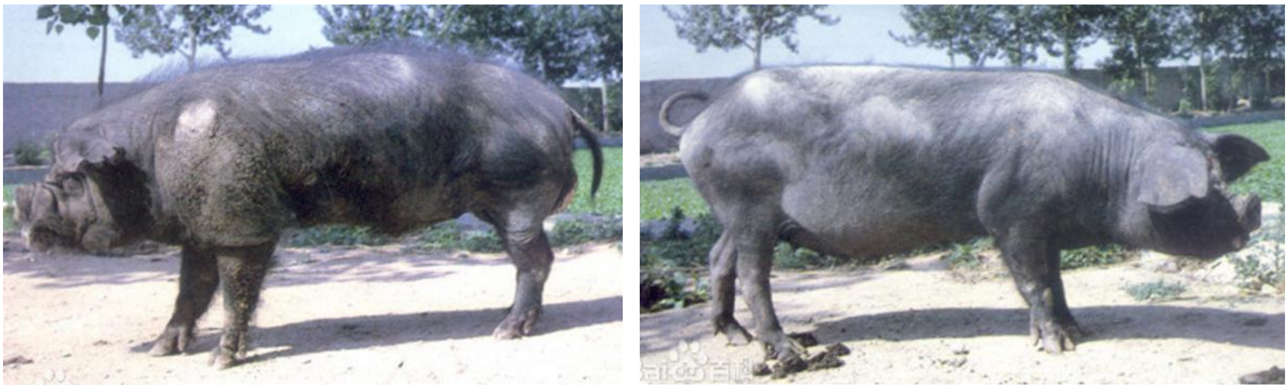

Figure S4. Pictures of Yimeng Black pigs (left:male; right:female) (Note: The pictures were taken from the website of https://baike.baidu.com/item/%E6%B2%82%E8%92%99%E9%BB%91%E7%8C%AA/731921?fr=aladdin.)

Yantai Black pigs

Yantai black pigs are distributed mainly in Yantai, Weihai and its surrounding areas. The representative photos for male and female Yantai black pigs are shown in Figure S5. They are of medium sized, with broad and wrinkled forehead, medium-sized and half drooping ears, and slightly pouted mouth. Their coat is black with moderate dense hair. Their buttock is plump, effective nipples are mostly 8 orderly arranged pairs. Compared with pigs of other Shandong indigenous breeds, the growth rate is fast and lean meat rate is high with average 50%. Sows of this breed have low prolificaty, with the average litter size 9.8.

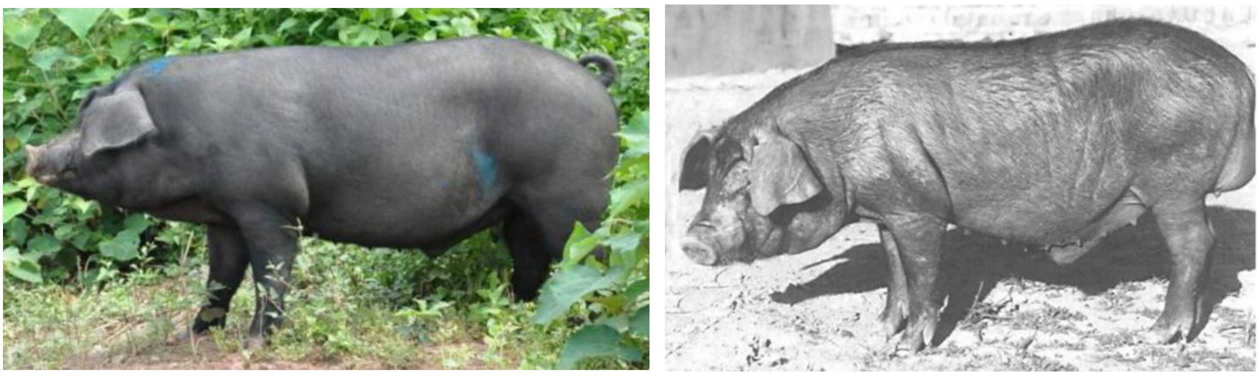

Figure S5. Pictures of Yantai Black pigs (left:male; right:female) (The pictures come from website of

https://baike.baidu.com/item/%E7%83%9F%E5%8F%B0%E9%BB%91%E7%8C%AA.)

Licha Black pigs

Licha black pigs are distributed mainly in Qingdao and its surrounding areas. The representative photos for male and female Licha black pigs are shown in Figure S6. They are of large sized, with strong constitution and symmetrical structure. Their heads are medium sized, with long face, broad and wrinkled forehead, and large and drooping ears. Their backs are straight, abdomen slightly droops, and buttocks lack of fullness. Effective nipples are mostly 7-8 pairs. Their lean meat rate is about 50%. Their most prominent characteristics is long body length due to the presence of 1-2 vertebras more than pigs of other Shandong indigenous pig breeds.

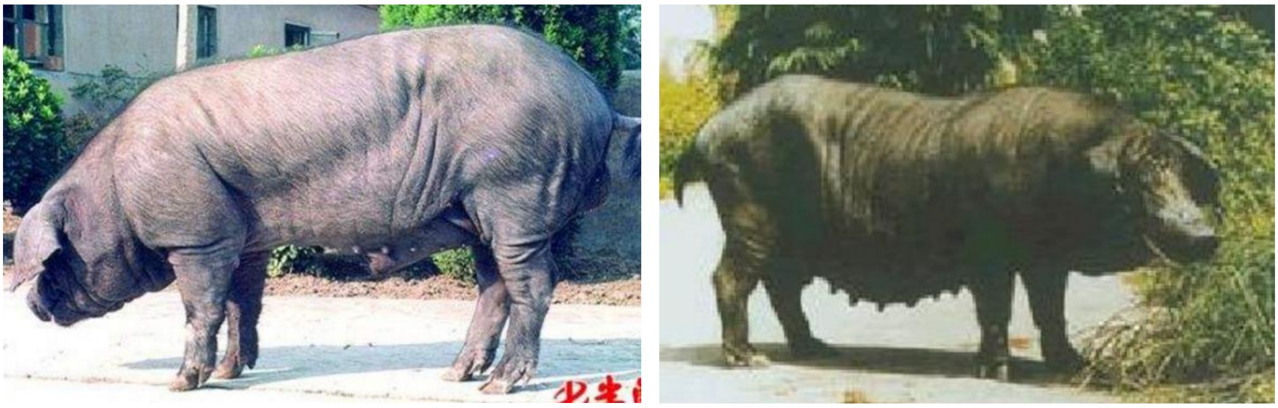

Figure S6. Pictures of Licha Black pigs (left:male; right:female) (The pictures were taken from the website of https://baike.baidu.com/item/%E9%87%8C%E5%B2%94%E9%BB%91%E7%8C%AA.)

Wulian Black pigs

Wulian Black pigs are distributed mainly in Rizhao and its surrounding areas. The representative photos for male and female Wulian Black pigs are shown in Figure S7. They are of large sized, with thick and long mouth and medium-sized and drooping ears. They have broad and rhomboid shaped wrinkles on the forehead. Their backs are straight, abdomens are large and compact, and buttocks are rather fullness. The sow's breast is well developed, with more than 7 pairs of effective nipples. The average lean meat rate is 50%, and the average litter size is 11. The most prominent characteristics of the breed is that they are suitable for house feeding and grazing, and have strong capability for digesting roughage.

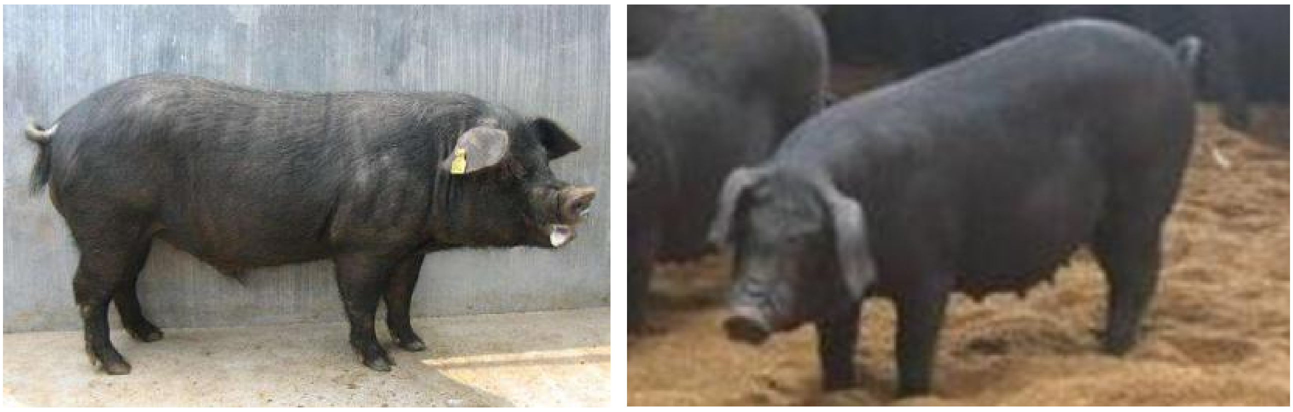

Figure S7. Pictures of Wulian Black pigs (left:male; right:female) (The pictures were taken from the website of http://www.rzda.gov.cn/newsview.aspx?tid=24&bid=101&cid=7284)

Table S1. SNP distribution and average distance of the adjacent SNPs on every chromosome

Chr.	SNP No.	SNP distance
1	4673	67.43
2	2467	65.88
3	2247	64.23
4	2228	64.41
5	1658	67.24
6	2481	63.52
7	2111	63.85
8	2270	66.43
9	2387	64.39
10	1142	68.88
11	1245	70.48
12	1022	62.20
13	3358	65.12
14	2448	62.85
15	2317	68.07
16	1338	64.97
17	1084	64.10
18	999	61.28
X	1825	78.86

Table S2. Number of SNP in different minimum allele frequency (MAF) ranges for each breed analysed

Breeds	0-0.05	0.05-0.1	0.1-0.2	0.2-0.3	0.3-0.4	0.4-0.5
BK	10926	3172	6173	6606	6064	6359
D	10263	3376	6804	6396	6491	5970
YK	5448	3365	6328	8178	7330	8650
PT	8959	3020	6184	7542	6811	6783
L	4086	3488	6083	8240	7944	9459
LW	13651	5394	6256	5271	4255	4472
DP	7498	6481	8516	5500	5549	5755
YT	2303	2425	6174	8169	9443	10786
LC	1954	2901	6767	8629	9787	9262
YM	5850	3099	6335	7344	8483	8188
WL	1591	2707	7181	8567	9201	10053
HG	4022	4994	7523	7554	7133	8073

Table S3. Linkage disequilibrium (r^2) levels at different distances for each breeds analysed

Distance (Mb)	Berkshire	Yorkshire	Duroc	Landrace	Pretriain	Laiwu	Dapulian	Yantai	Licha	Yimeng	Wulian	Heigai
0-0.01	0.6259	0.5799	0.6543	0.5363	0.6327	0.4508	0.4093	0.4613	0.4174	0.5202	0.4035	0.4141
0.01-0.02	0.5455	0.4988	0.5614	0.4503	0.5438	0.3882	0.3584	0.3949	0.3569	0.4484	0.3478	0.3603
0.02-0.03	0.5162	0.4609	0.5177	0.4161	0.5158	0.3438	0.3359	0.3632	0.3245	0.4226	0.3085	0.3222
0.03-0.04	0.4881	0.4397	0.5004	0.3954	0.4902	0.3395	0.3146	0.3446	0.3098	0.4062	0.2964	0.3105
0.04-0.05	0.4737	0.4335	0.4897	0.3798	0.4872	0.3409	0.3080	0.3331	0.3023	0.3992	0.2944	0.3094
0.05-0.1	0.4326	0.3969	0.4410	0.3464	0.4459	0.3197	0.2919	0.3046	0.2732	0.3630	0.2613	0.2795
0.1-0.15	0.3985	0.3650	0.4074	0.3127	0.4124	0.3000	0.2741	0.2792	0.2501	0.3385	0.2392	0.2642
0.15-0.2	0.3795	0.3438	0.3872	0.2890	0.3932	0.2923	0.2620	0.2622	0.2375	0.3233	0.2256	0.2535
0.2-0.25	0.3622	0.3269	0.3667	0.2752	0.3744	0.2834	0.2564	0.2505	0.2262	0.3107	0.2143	0.2446
0.25-0.3	0.3441	0.3118	0.3512	0.2596	0.3620	0.2766	0.2500	0.2409	0.2147	0.3021	0.2094	0.2369
0.3-0.35	0.3354	0.3039	0.3365	0.2518	0.3504	0.2690	0.2443	0.2338	0.2092	0.2935	0.2016	0.2314
0.35-0.4	0.3195	0.2939	0.3291	0.2414	0.3385	0.2658	0.2413	0.2247	0.2031	0.2870	0.1956	0.2261
0.4-0.45	0.3128	0.2806	0.3125	0.2320	0.3301	0.2626	0.2352	0.2196	0.1978	0.2802	0.1905	0.2236
0.45-0.5	0.3045	0.2781	0.3075	0.2233	0.3253	0.2570	0.2303	0.2165	0.1940	0.2761	0.1856	0.2193
0.5-0.55	0.3015	0.2638	0.2977	0.2162	0.3176	0.2552	0.2281	0.2110	0.1888	0.2708	0.1833	0.2170
0.55-0.6	0.2980	0.2611	0.2912	0.2115	0.3094	0.2493	0.2235	0.2074	0.1854	0.2640	0.1797	0.2122
0.6-0.65	0.2892	0.2519	0.2866	0.2045	0.3038	0.2480	0.2240	0.2053	0.1812	0.2623	0.1754	0.2133
0.65-0.7	0.2832	0.2478	0.2799	0.2033	0.2974	0.2451	0.2204	0.2011	0.1790	0.2594	0.1727	0.2096
0.7-0.75	0.2801	0.2470	0.2755	0.2001	0.2962	0.2393	0.2191	0.1966	0.1744	0.2539	0.1707	0.2071
0.75-0.8	0.2748	0.2402	0.2671	0.1954	0.2909	0.2358	0.2156	0.1947	0.1725	0.2515	0.1679	0.2069
0.8-0.85	0.2690	0.2350	0.2568	0.1905	0.2829	0.2348	0.2139	0.1920	0.1686	0.2467	0.1660	0.2044
0.85-0.9	0.2694	0.2303	0.2566	0.1897	0.2848	0.2324	0.2133	0.1896	0.1696	0.2457	0.1644	0.2052
0.9-0.95	0.2600	0.2234	0.2521	0.1821	0.2755	0.2292	0.2072	0.1856	0.1657	0.2391	0.1599	0.1992
0.95-1	0.2588	0.2184	0.2474	0.1788	0.2733	0.2288	0.2077	0.1841	0.1624	0.2360	0.1587	0.1992
Average of 5 Mb	0.2151	0.1778	0.1942	0.1555	0.2300	0.1949	0.1840	0.1589	0.1377	0.2012	0.1354	0.1790
Average of 1 Mb	0.3222	0.2869	0.3216	0.2391	0.3370	0.2630	0.2380	0.2263	0.2024	0.2846	0.1953	0.2278
adjacient SNP	0.4607	0.4249	0.4729	0.3891	0.4730	0.3379	0.3120	0.3318	0.2994	0.3888	0.2868	0.3048

Table S4. Estimated past effective population size (Ne) for each breeds analyzed

Generation (T)	Berkshire	Yorkshire	Duroc	Landrace	Pretriain	Laiwu	Dapulian	Yantai	Licha	Yimeng	Wulian	Heigai
13157.89	3931.88	4766.02	3475.75	5687.76	3818.57	8016.51	9494.04	7682.25	9182.94	6068.78	9726.43	9308.93
4385.96	1827.19	2203.75	1713.18	2677.42	1840.05	3455.40	3926.56	3360.80	3951.03	2697.93	4112.76	3893.12
2631.58	1233.05	1538.99	1225.83	1846.42	1235.27	2511.11	2601.06	2306.56	2738.42	1797.91	2949.49	2768.60
1879.70	985.57	1197.85	938.29	1436.87	977.47	1828.67	2047.97	1787.69	2093.86	1373.73	2231.14	2086.57
1461.99	812.04	955.17	761.69	1193.50	769.30	1413.49	1642.44	1463.60	1687.04	1100.34	1752.14	1631.37
877.19	575.32	666.44	555.90	827.48	545.05	933.13	1064.09	1001.33	1166.96	769.56	1239.77	1130.43
526.32	397.17	457.92	382.82	578.46	375.03	614.08	696.91	679.49	789.04	514.33	837.19	732.95
375.94	307.39	358.72	297.52	462.37	290.04	455.11	529.34	528.82	603.63	393.38	645.38	553.61
292.40	257.41	301.04	252.53	385.10	244.28	369.64	423.93	437.36	500.19	324.39	536.04	451.46
239.23	227.96	264.03	220.94	341.15	210.84	312.76	358.94	376.91	437.48	276.39	451.53	385.21
202.43	200.57	231.88	199.53	300.75	187.65	275.12	313.05	331.78	382.50	243.60	400.91	336.17
175.44	186.86	210.74	178.85	275.63	171.39	242.35	275.80	302.65	344.19	217.97	360.73	300.31
154.80	170.07	198.45	170.26	256.25	157.08	217.36	251.64	274.99	313.90	198.87	328.81	268.69
138.50	158.18	179.81	155.96	240.84	143.64	200.16	231.42	250.57	287.65	181.53	303.91	246.60
125.31	145.15	174.86	147.84	227.12	134.64	182.89	212.01	234.28	269.19	168.70	279.18	226.13
114.42	134.78	161.87	139.28	213.23	127.70	172.28	198.75	218.62	251.43	159.48	261.15	212.37
105.26	129.36	156.28	131.03	204.73	120.62	159.57	182.38	203.69	237.85	148.04	247.40	194.10
97.47	123.32	147.95	125.41	191.01	115.10	150.08	172.38	193.57	223.49	139.10	233.43	183.74
90.74	116.61	138.36	119.31	181.43	107.79	144.23	161.70	185.46	214.74	133.33	220.50	173.70
84.89	111.99	134.25	116.46	174.75	103.44	137.53	154.42	175.60	203.66	126.35	210.36	162.68
79.74	108.35	129.79	115.40	169.41	101.09	129.92	146.52	167.77	196.66	121.76	200.30	155.22
75.19	101.93	125.65	108.94	160.61	94.42	124.17	138.66	160.66	184.04	115.40	191.14	145.62
71.12	101.23	123.59	105.52	159.77	93.50	119.59	136.10	156.07	179.03	113.17	186.89	142.97
67.48	96.65	120.77	102.65	154.94	89.69	113.75	128.70	149.57	174.02	109.21	178.82	135.65

Table S5. Cross-validation (CV) error for each K value in historical admixture analysis

K	CV error
2	0.6446
3	0.61
4	0.58125
5	0.56418
6	0.55393
7	0.54144
8	0.53002
9	0.5208
10	0.514
11	0.51037
12	0.50904
